# G-quadruplex-mediated genomic instability drives SNVs in cancer

**DOI:** 10.1093/nar/gkae098

**Published:** 2024-02-26

**Authors:** Tilmann Richl, Jochen Kuper, Caroline Kisker

**Affiliations:** Rudolf Virchow Center for Integrative and Translational Bioimaging, University of Wuerzburg, Wuerzburg 97080, Germany; Rudolf Virchow Center for Integrative and Translational Bioimaging, University of Wuerzburg, Wuerzburg 97080, Germany; Rudolf Virchow Center for Integrative and Translational Bioimaging, University of Wuerzburg, Wuerzburg 97080, Germany

## Abstract

G-quadruplex (G4s) DNA structures have been implicated in inducing genomic instability and contributing to cancer development. However, the relationship between G4s and cancer-related single nucleotide variants (cSNVs) in clinical settings remains unclear. In this large-scale study, we integrated experimentally validated G4s with genomic cSNVs from 13480 cancer patients to investigate the spatial association of G4s with the cellular cSNV landscape. Our findings demonstrate an increase in local genomic instability with increasing local G4 content in cancer patients, suggesting a potential role for G4s in driving cSNVs. Notably, we observed distinct spatial patterns of cSNVs and common single nucleotide variants (dbSNVs) in relation to G4s, implying different mechanisms for their generation and accumulation. We further demonstrate large, cancer-specific differences in the relationship of G4s and cSNVs, which could have important implications for a new class of G4-stabilizing cancer therapeutics. Moreover, we show that high G4-content can serve as a prognostic marker for local cSNV density and patient survival rates. Our findings underscore the importance of considering G4s in cancer research and highlight the need for further investigation into the underlying molecular mechanisms of G4-mediated genomic instability, especially in the context of cancer.

## Graphical Abstract

**Graphical Abstract:**
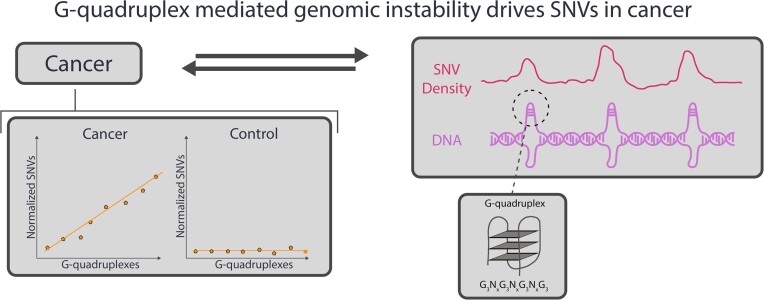

## Introduction

Next to the well-known B-DNA, there are many other non-B-DNA structures that can be adopted by DNA, such as hairpins, R-loops, triple helices, Z-DNA, A-DNA and G-quadruplexes (G4s) (1–3). Non-B-DNA structures can form in 13% of the human genome, with major implications for vital cellular processes (4).

G4s were first described *in vitro* by Sen and Gilbert in 1988, who identified single-stranded DNA sequences that self-associate to form parallel, four-stranded structures known as G-quadruplexes (5). They can form through π-π stacking of a minimum of two guanine quartets coordinating a sodium or potassium cation in the center, resulting in distinct four-stranded helical superstructures with high thermodynamic stability under physiological conditions. G-quartets consist of four planarly arranged guanines connected through cyclic Hoogsteen hydrogen bonds on the Watson-Crick and Hoogsteen interfaces, resulting in a total of four hydrogen bonds per guanine (6,7), thus differing from Watson-Crick base pairs of B-DNA, in which G-C pairs can only form three hydrogen bonds (6).

The overall topology of G4s is defined by the number of stacked quartets, the number of participating DNA strands, loop-types and -lengths, conformation of syn- and anti-guanines and groove sizes (6)—so far, the staggering amount of 27 different G4 topology classes have been described, which can be clustered in parallel-, antiparallel- and hybrid-G4s (6). Furthermore, both intra- and inter-molecular DNA-assemblies can give rise to G4s, leading to a high degree of topological polymorphism (8). Conventional intramolecular G4s can be described by the regex-motif G_*x*_N_*y*_G_*x*_N_*y*_G_*x*_N_*y*_G_*x*_, where *x* ≥ 3 guanosine residues and *y* is an interspacing loop with 1 ≤ *y* ≤ 7 nucleotides (8). Recently, also more flexible G4s with longer loops or different G-stacks have been observed (9,10).

G4-forming sequences can be found abundantly in the human genome: Early computational approaches predicted more than 300 000 potential G4-forming sequences in the human genome using the G4-consensus sequence (11,12). However, the development of computational methods capable of identifying G4s with more complex structural variants remains an ongoing challenge. (8)

Recent advances in experimental methods, such as G4 chromatin immunoprecipitation sequencing (G4 ChIP-seq), have significantly improved the ability to detect and investigate G4s in the human genome. G4 ChIP-seq employs chromatin immunoprecipitation with BG4 anti-G4 antibodies, followed by high-throughput sequencing (13). For methods such as G4-seq, sequencing is preceded by genome-wide stabilization of G4s using ligands like Pyridostatin (PDS), permitting the identification of G4s with improved accuracy and coverage (14). New antibody-agnostic approaches map G4s by digesting chromatin with nucleases or rely on small molecules to establish genome-wide interaction maps (15,16).

In addition, other methods have been developed to infer the presence of G4s through the mapping of G4-binding proteins. Examples of such proteins include the α-thalassemia mental retardation X-linked protein (ATRX) (17) and the xeroderma pigmentosum group B and D helicases, XPB and XPD (18). These proteins display high affinities for G4 structures and can serve as markers for their presence in the genome (10,18).

Intriguingly, the genomic localization of G4s is highly conserved between distinct species and over all domains of life (19) – indicating a high selection pressure to retain such sequences at specific genomic sites. G4 formation has been observed particularly in regulatory regions of chromatin, such as intron–exon-borders, telomeres, protein coding- and promotor regions and has been associated with transcriptional- and translational regulation and ribosome targeting - most human genes contain at least one G4 (20).

On a more granular level, the presence of G4s in the promoter regions of certain genes offers intriguing insights into their potential role in modulating gene function (21). The MYC, KIT and KRAS genes, all of which are implicated in various cancers, comprise G4-forming sequences within their promoters (22). In the case of the MYC gene, which is often deregulated in many solid tumors (23–25), the G4 within its promoter region has been shown to be a silencer element. This silencing effect can be enhanced by stabilization of the G4 with G4-stabilizing ligands, leading to a decrease in MYC expression. These observations suggest that G4-stabilizing ligands specifically targeting key oncogenes could serve as a potential therapeutic approach to combat cancer progression (22).

While G4s seem to function as a regulatory layer in eukaryotic cells, there is strong evidence that deregulated G4-formation is a major driver of genomic instability: *in-vitro* stabilization of G4s in human MRC5 fibroblasts with small ligands such as PDS resulted in cells arresting in G2-phase, mediated primarily through DNA damage checkpoint activation (26). Treatment with stabilizing G4-ligands was further shown to cause DNA double strand breaks and telomere maintenance problems *in vivo* (27–29). Consistently, aberrant G4-formation is linked to increased genome instability by facilitating mutations, deletions, and recombination events (27,28). Furthermore, G4s can form barriers for replication and transcription (30) and promote double-strand break formation (31–33). These effects are aggravated in cells lacking functional G4-resolving helicases, further strengthening the role of G4s as drivers of genomic instability (34–36). The inactivation of G4-resolving helicases, including BLM (Bloom syndrome) (37), WRN (Werner syndrome) (38), XPD (*Xeroderma pigmentosum*) (39), FANCJ (Fanconi anemia) (40), or PIF1 (41), results in serious pathologic phenotypes, all of which are invariably associated with an elevated predisposition towards cancer development (39,42).

G4s are known to promote genomic instability by interfering with the replication machinery: Through G4 formation on both the leading and the lagging strand, G4s can sterically block or stall replication forks, causing DNA double-strand breaks and ultimately resulting in increased mutagenesis (30). Once manifested, G4-conveyed DNA damage is passed on to daughter cells and thus can promote disease and evolution (30,43).

Another potential mechanism for G4-mediated genomic instability might involve the prolonged formation and stabilization of R-loops (27,44). In cancer cells employing a telomerase-independent telomere maintenance method, G4s and R-loops were found to be spatially linked and both engaged in an interdependent structure called G-loop (45). R-loop formation typically occurs during transcription in G-rich or purine-skewed regions (46), when the nascent RNA-template hybridizes with the template strand, leaving the non-template strand without a complementary binding strand (30). This can result in vulnerable single-stranded DNA-regions, which facilitate G4-formation and thereby further stabilize the single strand (27,47). Combined changes in the cellular G4 landscape may impact genomic integrity and the cellular transcriptome and may result in altered mutational signatures.

G4s can also modulate the formation of somatic structural variants in cancer cells (48). Furthermore, larger deletions and complex chromosomal rearrangements could be linked to aberrant G4-formation (27). In prostate cancer patients, intracellular G4-levels could be correlated with cancer progression and progression-free survival (49). These data indicate that G4-associated mutations can confer selective advantages to malignant cells and ultimately might drive cancer development.

Cancer is fundamentally driven by the accumulation of genetic mutations and genomic instability, and thus G4s may act as facilitators of cancer development. With the suggested role of G4s in cancer development and progression, the impact on genomic stability has emerged as a promising target for new therapeutic approaches. However, further understanding of possible mechanisms that drive G4-mediated cancer progression are crucial for the development of effective treatments.

One type of genomic instability that can drive the development of cancer are single nucleotide variants (SNVs). SNVs are point mutations resulting in single base pair changes that occur at a specific position in the DNA sequence. They are the most common type of genetic variation in the human genome and can have a range of functional effects, such as modifying gene expression, the function of the encoded protein or altering the stability of non-B-DNA structures (50). SNVs within a gene or in a regulatory region near a gene may play significant roles in disease development and progression. In the context of cancer, single point driver mutations can often cause or aggravate tumor growth and progression (51).

In this study, we conducted an analysis of the spatial association between G4s and SNVs in a large cohort of 13.480 cancer patients encompassing multiple cancer types. Our results indicate a significant positive correlation between the local densities of G4s and SNVs in cancer patients, suggesting a potential link between G4-mediated genomic instability and the accumulation of somatic mutations. Additionally, we observed variations in the strength of this correlation among different cancer- and tissue types, highlighting the heterogeneity of the SNV landscape in cancer. Our study thus provides novel insights into the role of G4s in the genomic variability and instability of cancer that demonstrate their importance as prognostic markers and potential target structures.

## Materials and methods

### Data collection and processing

#### Collection of genomic data

We obtained cSNVs from human cancer samples available in the open-access National Cancer Institute GDC Data Portal (52) (https://portal.gdc.cancer.gov). Specifically, we included all 14 932 whole-exome sequencing experiments of the single nucleotide variation data category, which initially yielded 3 454 377 cSNVs from 13 480 patients. The experiments were derived from various programs, including TCGA, CPTAC, MMRF, BEATAML 1.0, TARGET, CMI, HCMI, CGCI, CDDP_EAGLE and EXCEPTIONAL_RESPONDERS. The collected data were downloaded and processed using a custom shell script and analysed using a custom Python script.

We collected common dbSNVs from the UCSC genome browser (https://genome.ucsc.edu, Version 151) and processed them with a custom shell script. In total, 15 175 044 dbSNVs were considered in this analysis.

#### Human genome collection

The human GRCh38 genome was obtained from the National Institutes of Health (NIH, https://www.ncbi.nlm.nih.gov/).

#### Pre-processing of the data

To ensure statistical reliability, we removed known germline mutations by intersecting cSNVs with common SNVs and excluded cancer types with less than 10 samples. Ultimately, we considered 2 847 440 cSNVs for this study.

#### G4-quadruplex data

We used experimentally validated G4s in our study to ensure a high level of confidence in our results. The genomic locations of G4s used in this study were obtained from Balasubramanian et al. (10). We converted the G4 positions to the hg38 assembly using the UCSC LiftOver tool (https://genome.ucsc.edu/cgi-bin/hgLiftOver).

In total, 716 310 G4s were considered in the human genome. To ensure the reliability of the G4 data, we excluded genomic windows with more than 15 G4s due to statistically insufficient window quantities.

#### Random G4-like sequences generation

Random G4-like sequences were generated in human exons with the bedtools random function from the bedtools suite (53) and raised flags -l 200 -n 50000000.

#### Cancer genes

The Cancer Gene Census (CGC, https://cancer.sanger.ac.uk/census) was used to identify known cancer genes. A total of 736 genes were selected based on their documented activity or strong indications of involvement in cancer.

#### PQS-prediction

PQSs were predicted in the human hg38 genome using pqsfinder (54) v2.0.1, with default algorithm options. In total, 1 352 359 potential G4-forming sequences were predicted, of which 828 901 were located within the human exome.

### Genome-wide spatial correlation

#### Spatial correlation

For each cSNV or dbSNV or randomized SNV, the absolute distance in base pairs to the next G4 was calculated using the bedtools closest function with raised flags -d. Then, local densities at G4s were calculated using a custom python script.

#### Parametric Gaussian Fitting

We utilized a custom python script to employ Gaussian distribution fitting to quantitatively assess and compare the observed distributions of cSNVs or dbSNVs at G4s. By comparing the coefficients of the fitted Gaussian distributions, we were able to evaluate the goodness-of-fit and determine the relative similarity or difference between the distributions of cSNVs or dbSNVs at G4s.

### Genomic window analysis

#### Fragmentation of the genome into fixed-end or sliding windows

We retrieved annotated genes from the GENCODE V43 database and limited genomic windows to encompass genes plus the 2 or 5 kb flanking region. This area was then fragmented into sliding windows of 5 or 50 kb and a step size of 1 kb with the makewindows function from the bedtools suite (53) and flags -w 5000 -s 1000, or into fixed-end windows of 5 kb with the makewindows function from the bedtools suite and flags -w 5000. All genomic windows, where the distance between exons exceeded the window size, were excluded. For the statistical analyses, only G4s and cSNVs or dbSNVs inside exonic sequences in genomic windows were considered.

#### Local cSNV or dbSNVs enrichment analysis

5 kb genomic windows were sorted into groups containing either zero- (0), non-zero- (1+) or ten-or-more (10+) G4s or stratified based on the number of G4s contained. Then, the median and mean number of cSNVs or dbSNVs was determined for each group. Significance levels were calculated using either the indirect t-test or the chi-squared test.

#### Density and correlation of G4s and cSNVs in single genes

Fasta-sequences of coding exons of the CDK4 (ENST00000257904.11) and MYC (ENST00000621592.8) genes were obtained for the human hg38 genome from the GENCODE 44 database and converted to BED-format using a custom awk-script. Coding exons were intersected with G4s and cSNVs using the intersect function from the bedtools suite (53). For correlation analysis, a custom python-script was used to normalize cSNV-density to cSNVs / bp per coding exon and correlate the cSNVs / bp with the number of G4s per coding exon.

#### G4-content of genes

Genes were divided into cancer genes and non-cancer genes, based on genes classified as cancer genes by TCGA. The median and mean number of G4s was assessed for both groups using a custom python script.

#### Analysing cSNVs or dbSNVs in cancer- or non-cancer genes

Genes were divided into cancer genes and non-cancer genes as described before. For both groups, the density of cSNVs or dbSNVs in genomic windows with either zero or ten-or-more G4s was calculated using a custom python script.

#### Incomplete categorical bootstrapping

Genomic windows were stratified based on the number of G4s they contained as categorical variables. To account for the significant disparities in the sample sizes among different categorical variables, *N* permutations (250/500/1000) of *I* sample points (1–100) were randomly selected from each category - for each permutation, the bootstrapped *I* sample points were reduced to a single average value. The thereby generated, random-sampled subset consisted of N * I datapoints and was used to train a linear regression model. This approach was repeated for the number of cycles in parentheses (10/20/50) and *I* was incremented by 1/5/10 (*i*) for each incrementation.

#### Linear regression modelling

We trained the linear regression models using the linear model class from the python package sklearn (version 1.2.1) with default parameters. For data preparation, the human GRCh38 genome was fragmented into 358 750 non-overlapping 5 kb windows. For every window, the number of cSNVs, dbSNVs and G4s was recorded. Linear regression models were either trained with the complete data available or limited to subsets of data generated through incomplete categorical subsampling. *R*^2^-values und linear regression coefficients were determined for each model. Wherever we tested the linear regression models, data were randomly split into 70% training data and 30% test data.

### Tissue- and cancer-specific associations

#### Computation of the normalized G4-average

For each patient, all genomic 5 kb windows containing at least one cSNV were selected and the mean number of G4s across these windows was determined. This number was then normalized to the sum of G4s across the selected genomic windows.

#### Computation of the cSNV-average

For each patient, all genomic 5 kb windows containing at least one cSNV were selected and the mean number of cSNVs across these windows was determined.

#### Intra-cancer variance

Patients were grouped based on their primary diagnoses. For each patient, the G4-average was determined. The normalized variance within one patient-group was then calculated by dividing the group standard deviation by the group mean.

### Survival regression

#### Fitting Cox's proportional hazards model

We selected 3416 patients which had passed away due to cancer-related causes or remained alive throughout the individual data collection period. For each patient, the global G4-average and the global cSNV-average was determined and used as predictor variables to fit Cox's proportional hazards model, implemented in the lifeline package (55). Significance values, hazard coefficients and model concordance were drawn from the models’ provided functions.

### GC normalization

#### Normalization to local GC content

The G4-content in each fixed-length genomic window was normalized to GC count by dividing the number of G4s in each genomic window by the number of GC-base pairs per window, yielding G4s/GC-base pair for each window.

#### Assessing the partial correlation

Partial correlation between the number of G4s per window and the number of cSNVs per window was calculated using the pingouin package in python (Version 0.5.3) and controlling for the GC-content as a fraction of base pairs in each genomic window.

## Results

### SNVs are spatially correlated with G4s

It has been shown that the stabilization of G4s with small ligands, such as PDS, can increase genomic instability and induce cellular DNA damage response (DDR) pathways, ultimately leading to cell cycle arrest in the G2 phase (26). Given the frequent occurrence of G4 formation in cancer tissue and recent evidence linking G4s to mutation hotspots in lymphoma cells (56), we investigated the genome-wide spatial correlation of SNVs with G4s.

**Figure 1.**
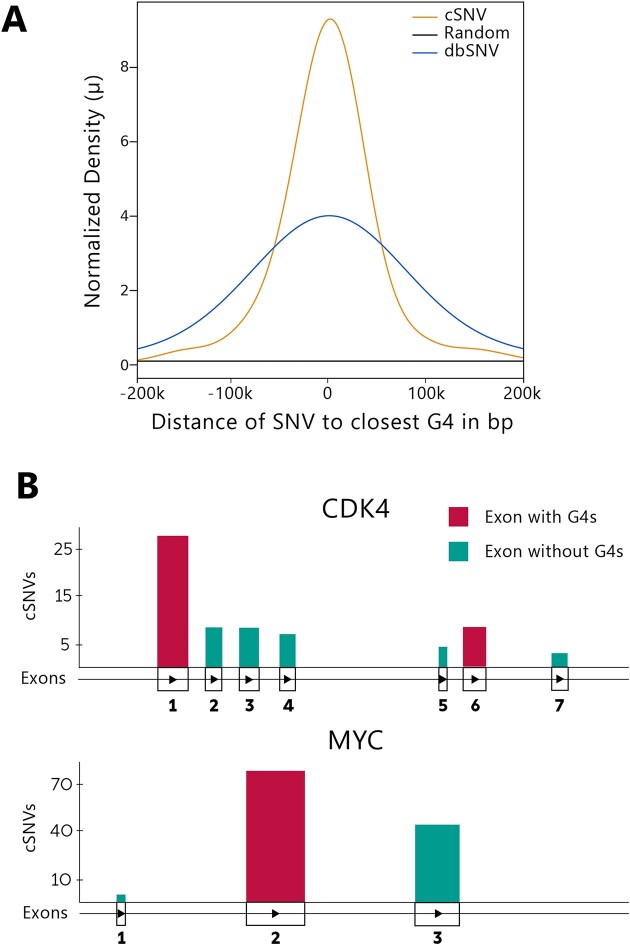
Genome-wide spatial correlation of cSNVs or dbSNVs with G4s. (**A**) Normalized density distribution of cSNVs (orange), dbSNVs (blue) or random SNVs (black) at G4s. (**B**) Coding exon mutation frequency in relation to G4 presence for the *CDK4* and *MYC* genes. Bar width corresponds to exon length.

We collected SNVs in human exons from 13 480 cancer patients (cSNVs) with 154 different cancer types, obtained from the National Cancer Institute (https://portal.gdc.cancer.gov/). We excluded cSNVs that overlapped with known germline mutations in the Common SNV database, which consists of more than 15 million SNVs (dbSNVs) with a minor allele frequency >1% (57). We then calculated the absolute distance of each G4 to the nearest cSNV or dbSNV in base pairs and compared these distances to those of 50 million random G4-like sequences with similar average length (Figure 1A). This comparison aimed to determine if there was a significant spatial correlation between cSNVs or dbSNVs and G4s compared to random G4-like sequences.

While no significant enrichment of cSNVs or dbSNVs at random G4-like sequences was observed, both cSNVs and dbSNVs displayed a robust spatial correlation with G4s in human exons. Employing parametric Gaussian fitting to analyze the distances between cSNVs and dbSNVs to adjacent G4s, we identified differential 95% confidence intervals (CIs). Specifically, dbSNVs were predominantly located upstream of G4s, with a 95% CI ranging from –2143 to –447 bp. Conversely, cSNVs were located more proximally downstream of G4s, with a 95% CI spanning 63–391 bp. This distinction was further emphasized by the narrower CI of cSNVs, which was reflected in a 5.2-fold compressed Gaussian fit compared to dbSNVs, signifying a stronger spatial correlation between cSNVs and G4s.

Based on these results, we analyzed the linear relationships between cSNVs or dbSNVs and G4s by calculating the quotient of their covariance and the product of their respective standard deviations. Consistent with the earlier findings from Gaussian fitting, the Pearson correlation between cSNVs and G4s was 2.4-fold stronger (Pearson's *r*: 0.194) than the correlation between dbSNVs and G4s (Pearson's *r*: 0.082).

To determine whether the identified genome-wide association between cSNVs and G4s extended to individual genes, we analyzed the frequency distribution of cSNVs and G4s in a set of 736 hallmark cancer genes, which were selected based on established functional relevance or compelling evidence of involvement in oncogenic processes, as defined by the Cancer Gene Census. These genes are widely recognized as crucial players in the pathogenesis of cancer (Figure 1B). We found that the normalized density distributions of cSNVs and G4s were frequently corresponding and equally clustered in similar exons of these genes, with a significant positive correlation (Pearson's *r* = 0.243, *P* < 0.001). For example, in the CDK4 gene, a known oncogene that is mutated in a wide range of cancer types, we observed a high density of cSNVs in the coding exons one and six, which are the only coding exons harboring G4s in the CDK4 gene. Similarly, in the MYC gene, most cSNVs are located in the second coding exon, which is also the only coding exon to contain G4s. (Figure 1B). Normalizing the number of cSNVs per coding exon to the exon length resulted in moderate correlations with the number of G4s in coding exons (CDK4 Pearson's *r* = 0.505, MYC Pearson's *r* = 0.569). These findings suggest that the observed correlation between cSNVs and G4s is present both genome-wide and on an intragenic level.

Overall, our analysis suggests a strong spatial correlation between cSNVs and G4s in cancer patients, as well as a weaker but still significant correlation between dbSNVs and G4s.

### High G4 content is associated with local genomic instability

Building on the previously established genome-wide spatial correlation between cSNVs and G4s, we aimed to investigate this spatial correlation at a more granular scale. We therefore fragmented the genome into fixed-end 5 kb windows and analyzed the cSNV- or dbSNVs - and G4-count in each window. By comparing windows with zero G4s, non-zero G4s (i.e. at least one G4), and ten or more G4s, we can better assess whether the presence and quantity of G4s in a window has an impact on cSNV density. We excluded genomic windows with more than 15 G4s due to statistically insufficient window quantities.

**Figure 2.**
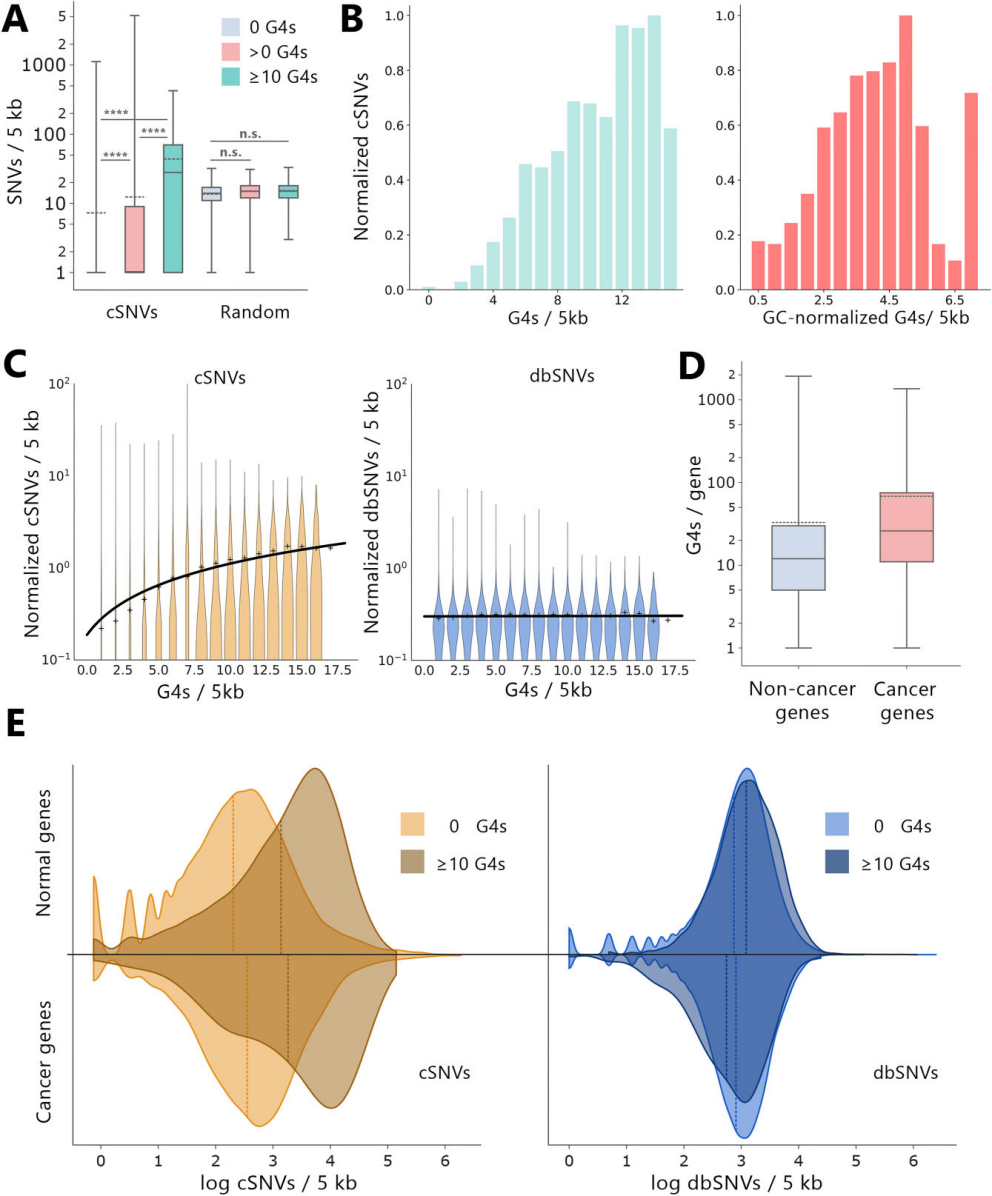
High local G4-content is associated with local genomic instability. (**A**) Average number of cSNVs or random SNVs in genomic windows containing either zero, non-zero or 10 or more G4s. (**B**) Average distribution of cSNVs in relation to G4s or GC-normalized G4s in 5 kb genomic windows. (**C**) Normalized density of cSNVs or dbSNVs in genomic windows stratified for the number of G4s contained in the respective windows. Means of the respective groups are indicated as plus markers, the black line represents a linear fit of the group means. (**D**) G4-content of non-cancer or cancer genes. (**E**) Density distribution of cSNVs or dbSNVs in 5 kb genomic windows with zero or more than ten G4s in either non-cancer or cancer genes.

Our results show that genomic windows without G4s contained significantly (ind. *t*-test = –44.238, *P* < 0.001) less cSNVs (mean: 6.5 cSNVs, median: 0 cSNVs) compared to genomic windows containing at least one G4 (mean: 10.8 cSNVs, median: 0 cSNVs). In contrast, there was no significant difference in cSNV-content across genomic windows with zero, non-zero, or more than ten random G4-like sequences in a simulated genome containing 50 million random G4-like sequences (Figure 2A). Moreover, in windows containing a large (≥10) number of G4s, the local number of cSNVs was on average 7.6-fold increased (mean: 49.5 cSNVs, median: 28 cSNVs, ind. *t*-test = –89.601, *P* < 0.001) compared to windows without G4s, indicating that the effect is G4-dependent. This observation remained consistent for fixed-end 20 kb- and 50 kb sliding-windows (data not shown).

To ensure that the observed increase in cSNVs is not caused by higher GC content, we normalized G4s per window relative to the internal GC count of each window. In line with our earlier findings, we observed a corresponding significant increase of cSNVs with increasing GC-normalized G4s per window, indicating that the local increase in cSNVs can be attributed to G4s rather than the GC-content (Figure 2B). Next, we calculated the covariance to quantify the correlation of G4s and cSNVs while controlling for GC content. Our results show a significant correlation of G4s and cSNVs in the absence of GC-content (*P*-value: 0), with only a slight decrease in Pearson's *r* from 0.173 to 0.158. Taken together, these results further indicate that the increase in cSNVs is largely driven by the presence of G4s rather than GC content.

To further explore the association between the G4 abundance in a genomic window and the degree of local genomic instability, we stratified genomic 5 kb windows based on the G4-count in each window. In this context, we considered local cSNV or dbSNV enrichment as marker for local genomic instability, as an increased frequency of SNVs can disrupt the normal function and regulation of genes, potentially leading to the development and progression of cancer (58).

We observed a robust linear increase in local normalized cSNV-count with increasing G4 content in cancer patients (Figure 2C). This effect was exclusively linked to cancer patients, as increasing G4-levels in genomic windows with common dbSNVs did not result in an increase.

Given that G4s were reported to cluster in functional regions and are enriched in genes involved in cancer formation (59), we hypothesized that cancer-specific genes might contain higher G4-levels, thus explaining the observed exclusive correlation in cancer patients. Comparing the average number of G4s in cancer or non-cancer genes, we found length-normalized cancer genes to contain a median average of 15 G4s per gene (mean: 27), while non-cancer genes contained a median average of 6 G4s per gene (mean: 15). Overall, the G4-levels were 2.8-fold increased in cancer genes compared to non-cancer genes (Figure 2D).

To account for higher G4-levels in cancer genes, we compared local cSNV- and dbSNV-levels in 5 kb genomic windows with either zero G4s or more than 10 G4s in cancer genes or non-cancer genes. We observed that there is a significant increase in cSNV-levels in windows with 10 or more G4s compared to windows without G4s (ind. *t*-test = 142.3, *P* = 0.0; chi-squared-test = 0.0), as indicated by a visible right-shift in the kernel-density-estimated probability curves (Figure 2E). This observation is consistent in both cancer genes and non-cancer genes. However, consistent with earlier results, the difference in dbSNV-levels for varying G4-levels is less pronounced and not significant in both cancer- and non-cancer genes.

Overall, our findings demonstrate a strong association between G4 content and local cSNV enrichment, indicating that G4s contribute to localized SNV-mediated genomic instability in cancer patients at a more granular scale.

### G4s are prognostic markers for cancer-related mortality and show distinct associations with cSNVs in various cancer- and tissue types

We sought to quantify the relationship between G4s and cSNVs in cancer patients and investigate possible differences across different cancer types. However, given the inherent heterogeneity of cancer, quantifying the observed correlation posed a significant challenge due to the high degree of variability among the samples and different cancer types. When stratifying cSNVs based on G4s per genomic 5 kb window, the intra-group variation was often larger than the inter-group difference, making it challenging to establish a clear relationship.

**Figure 3.**
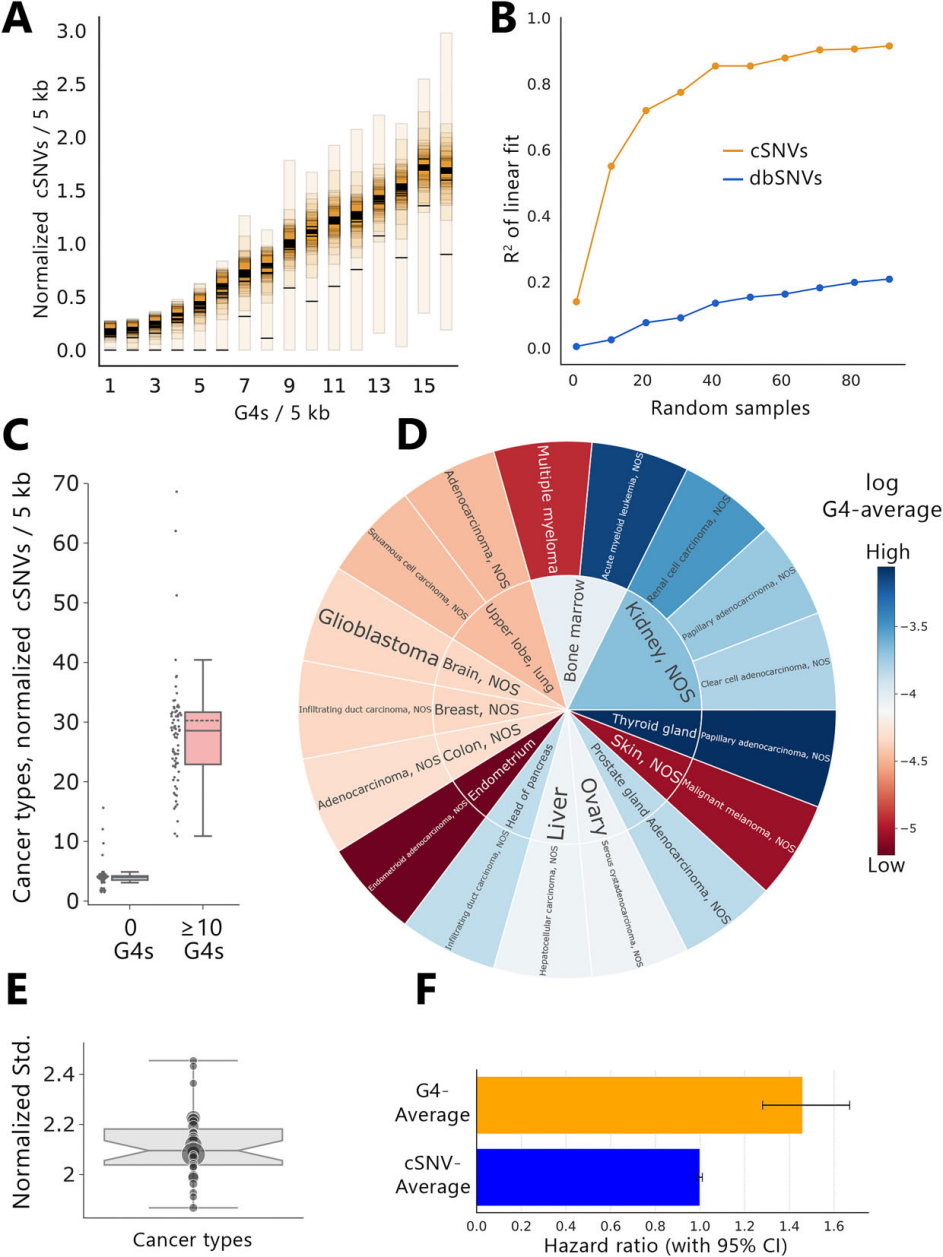
G4s show distinct associations with cSNVs in various cancer- and tissue types and could serve as prognostic markers for cancer-related mortality. (**A**) Incomplete categorical bootstrapping of cSNVs stratified for G4 content in genomic 5 kb windows. Black lines indicate the group mean. (**B**) *R*^2^ of the linear fit for increasing random sampling sizes. (**C**) Average normalized cSNVs for genomic 5 kb windows with either zero or more than ten G4s, grouped by cancer types. (**D**) Mean G4-average of patients across different tissue- and cancer types. (**E**) Mean-normalized standard deviation of G4-averages of patients with identical primary diagnoses. (**F**) Predictor variables G4-average or cSNV-average of Cox's Proportional Hazards Model.

To address this issue, we employed incomplete categorical bootstrapping. With this approach we aimed to quantify the genome-wide relationship between G4s and cSNVs in various cancer types by reducing the impact of sample size disparities and intra-group variance (Figure 3A).

In short, we stratified genomic windows based on the number of G4s. For each genomic window group, representative subsets of cSNVs were created through random sampling. Random-sampled points were incrementally averaged to reduce the intra-group variance.

Ultimately, by incrementally increasing the number of averaged random samples, we were able to fit a linear regression model on cSNVs, approximating an *r*^2^ of 0.96. This value indicates that 96% of the variance in the dataset can be explained by the linear relationship between cSNVs and G4s and demonstrated the strong correlation between G4s and cSNVs in cancer patients. Consistent with our prior findings, we could not approximate a significant linear correlation for common dbSNVs (Figure 3B).

Significant differences were observed in the strength of the correlation between G4s and cSNVs among different cancer- and tissue-types. Therefore, we aimed to further elucidate the tissue- and cancer-specific associations between G4s and cSNVs.

To study the relationship between cSNVs and G4s in greater detail, we calculated the average number of G4s found in 5 kb sections of the genome that contained at least one cSNV, for each cancer type within each tissue. This average is referred to as the G4-average. To make it easier to compare different cancer types with varying mutational profiles, we adjusted the G4-averages by dividing them by the total number of G4s found in all genomic sections with observed cSNVs for each cancer type, yielding the normalized G4-average.

When comparing the normalized cSNV counts in genomic windows containing either zero, non-zero or ten or more G4s, we found that cancer-specific disparities in cSNV distribution were primarily limited to genomic windows with G4s, particularly in those with ten or more G4s. In these regions, the most significant differences in normalized cSNV counts occurred across various cancer types such as multiple myeloma or cholangiocarcinoma. Furthermore, all cancer types displayed an increase in normalized cSNVs in genomic windows with 10 or more G4s compared to windows without G4s. Conversely, in genomic windows without G4s, normalized cSNV counts remained consistent across different cancer types, indicating that cancer-specific changes in cSNV density are limited to genomic regions harboring G4s (Figure 3C).

Our results reveal substantial tissue- and cancer-specific disparities in the global normalized G4-average of genomic windows containing cSNVs. For instance, cSNVs of patients suffering from acute myeloid leukemia consistently appeared in sections of the genome with a high normalized G4-average of 7.3, while other cancer types showed more varied associations. Some cancers, like malignant melanoma (normalized G4-average: 0.08) or multiple myeloma (normalized G4-average: 0.12), displayed consistently low normalized G4-averages in the genomic sections where cSNVs were found, across patients with these diagnoses. The difference between the highest and lowest G4-averages across all cancer types was substantial, spanning three orders of magnitude (Figure 3D).

Interestingly, we found that some tissues were primarily linked to cancer types with high G4-averages, hinting at a potential tissue-specific effect on the distribution of cSNVs in cancer-related genomic regions involving G4 structures. Cancers that originated from tissues like bone marrow, thyroid and the adrenal glands consistently displayed a higher G4-average.

After examining differences in G4-averages between various cancer types, we further explored variations within the same cancer type by grouping patients based on their primary diagnosis. We assessed the differences in G4-averages within these groups, using the mean-normalized standard deviation as an indicator of variance. Our analysis showed that the variations within each cancer type were relatively consistent across different cancer types, with a median standard deviation of 2.1 times the mean and an interquartile range (IQR) of 0.136.

Interestingly, the differences within each cancer type were often more substantial than the differences observed between distinct cancer types. This suggests that two patients with the same cancer diagnosis might have greater disparities in their G4-averages than patients with different cancer types (Figure 3E). This finding highlights the complex nature of cancer and the importance of considering individual variability when studying the relationship between G4s and cancer (Figure 3E).

With our observations on the highly significant association of cSNVs and G4s in cancer patients, we lastly aimed to investigate a potential function of the global G4-average in genomic windows with cSNVs as a prognostic marker for cancer patient outcomes. Based on the associated metadata, we selected 3416 patients that either had died due to their primary cancer diagnosis or remained alive throughout the data collection period. Patients with incomplete survival data or survival status, as well as patients who had died from causes unrelated to cancer were excluded. We calculated the global G4-average for each patient in genomic 5 kb windows containing cSNVs and utilized these data to fit Cox's proportional hazards model—a well-established model to investigate the association between the survival time of patients and predictor variables (60,61).

Our analysis revealed that the G4-average serves as a significant prognostic marker (*P* < 0.005) with a relative hazard coefficient of 0.38. This implies that for each one-unit increase in G4-average, the hazard of cancer-related death increases by 38%, associating high G4-averages with a worse prognosis (Figure 3F). In contrast, there was no significant impact of the individual cSNV count on the survival rate.

In summary, our findings demonstrate a substantial variation in the association of G4s and cSNVs across different cancer types and tissues, suggesting a possible tissue-specific influence on the G4-associated distribution of cSNVs in cancer-related genomic regions. Additionally, we identify the patient-specific global G4-average as a potential prognostic biomarker associated with poor prognosis.

## Discussion

Cancer is a disease fundamentally driven by the accumulation of genetic mutations and genomic instability. Specific high-impact mutations can result in the activation of oncogenes, inactivation of tumor suppressor genes, and perturbation of key cellular pathways (62,63). These alterations can confer selective advantages to malignant cells, such as enhanced proliferation, survival and metastatic potential, and thus ultimately drive cancer development (64).

Recent studies have demonstrated the involvement of G4s in inducing or aggravating genomic damage, such as copy number alterations, deletions or translocations (65). Further reports demonstrated that G4s cause replication-dependent DNA-damage or change the overall chromatin structure (66,67). While G4s have been predominantly associated with the promoters of oncogenes such as MYC, KIT or KRAS (23–25), where their stabilization is associated with suppressed oncogene expression, we explored their potential role in exonic regions and the associated impact on genomic instability in cancer patients. In the context of exonic regions, the presence and stabilization of G4s may contribute both to genomic instability and potentially influence cancer development by introducing cSNVs or by sterically hindering the transcription process. Though this is a less explored facet of G4 function, it may hold significant relevance given the intricate role of exons for proper genomic function.

Moreover, the application of G4-stabilizing ligands has been observed to activate DDRs and arrest cells in G2-phase, hinting at an increase in genomic instability due to aberrant G4 formation (30). Recent genome-wide investigations underscore that these effects are not confined to promotors, but are rather observed across the genome (68–70). In line with these findings, our study suggests that G4-mediated genomic instability could also be significant in exonic regions, potentially impacting protein function and stability rather than transcriptional regulation.

Depending on the magnitude, G4-mediated genomic instability can be both beneficial or detrimental for cancer formation: Similar to mutagenic cancer therapeutics or radiation therapy, massive DNA damage has been shown to induce cellular apoptosis and autophagy in tumor cells. One study has linked cellular treatment with G4 ligands, such as PDS or 20A, to anti-tumorigenic effects and G2/M cell cycle arrest (71). This effect seems to be aggravated in cells with an impaired DNA repair machinery and deficiencies in homologous recombination. (72).

However, while there is a substantial number of studies investigating the potential role of G4s as drivers of genomic instability, most of these studies remain confined to cellular models. Consistently, current knowledge regarding the effects of G4s on cancer development in a clinical context is limited.

Here, we present a large-scale study to investigate the spatial association of G4s with the cellular cSNV-landscape in the clinical context of cancer to shed light into the role of G4s in genomic instability and mutagenesis in cancer patients. To address this, we integrated experimentally validated G4s with genomic cSNVs from 13 480 cancer patients to investigate a potential relationship of G4s with primary cSNVs.

Our results show that with an increasing number of local PQSs there is increasing local genomic instability (as represented by high local cSNV content) in cancer patients. We observed that G4s, cSNVs and dbSNVs colocalize on a genomic scale. The density distribution of both cSNVs and dbSNVs is maximized in direct proximity of G4s. Interestingly, the 95% confidence intervals for neither cSNVs nor dbSNVs extend to the locations of G4s, with cSNVs predominantly located downstream and dbSNVs predominantly located upstream of G4s. Importantly, our results show that the increased density of cSNVs in the flanking regions of G4s cannot be explained by the GC-content, as we observed a qualitatively similar increase in cSNVs in genomic windows with high GC-normalized G4s. Furthermore, the correlation between cSNVs and G4s still remained highly significant after controlling for GC-content, with only a minor decrease in the Pearson's correlation coefficient. This underscores the potential influence of G4s on cSNV distribution, independent of GC-content.

Recent studies investigated the connection between G4s and SNVs, primarily focusing on SNVs within G4-motifs and the impact of these G4-variants on genetic regulation and disease (73–75). Furthermore, SNVs were reported to be generally enriched in G4-motifs and their immediate flanking regions (4,73). However, G4s have not yet been suggested as global drivers of cSNVs in cancer patients. Our study emphasizes a different perspective on the relationship between G4s and SNVs in cancer patients, revealing that most cSNVs are located in the 5 kb flanking regions of G4s rather than within the G4-motifs themselves, with only 12% of cSNVs being located within exonic G4-motifs. While this is still a significant number, this highlights the unique density distribution of cSNVs in close proximity to, but not within, G4s.

An important limitation of our study is that we correlated local PQS rather than formed G4s with cSNVs – thus, we cannot establish causality between G4s and cSNVs. Nevertheless, recent studies have shown that G4s can be detected in patient-derived tissues using immunohistochemistry, with a significantly elevated number of G4-positive nuclei observed in human cancers of the liver and stomach compared to non-neoplastic tissue (76). Furthermore, the prevalence of G4s was found to be significantly increased in immortalized cells as compared to primary human cells (13). This suggests that elevated G-quadruplex formation might be a characteristic of some cancers and supports the hypothesis that G4s could contribute to genomic instability in cancer patients (76). Although the directionality of the relationship between G4-mediated genomic instability and tumorigenesis cannot be inferred from our findings, our ability to predict patient survival rates based on G4 content provides strong evidence that there is likely at least a partial causal relationship between G4s and genomic instability in cancer development.

While this study provides a broad overview of the association between cSNVs and inferred G4s in cancer, it is beyond the scope of this manuscript to extend these analyses to an individual patient level. However, we suggest that future studies could explore this correlation in individual patients, utilizing both patient-specific cSNVs and chromatin-mapped G4s. This approach could serve to further elucidate the patterns and mechanisms we have discussed, as well as to investigate whether the observed trends can also be observed at the level of individual patients. Such an analysis could significantly enhance our understanding of the role of G4s in tumorigenesis and genomic instability.

One of the most intriguing aspects of our observations was the disparity between cSNVs and dbSNVs with respect to their association with G4s. Although there was a weaker but still significant genome-wide spatial correlation between G4s and dbSNVs, there was no local increase in dbSNVs observed in genomic windows with increasing G4-content. This suggests that there is a distinct cSNV landscape in cancer patients compared to dbSNVs, which could partly originate from their similar pathogenetic backgrounds. The observed distinct spatial patterns of cSNVs and dbSNVs in relation to G4s imply that different mechanisms may underlie their generation and accumulation. However, our results exclude the higher G4-content in cancer genes as origin of this effect, as this observation prevailed even after normalizing the data to cancer genes. Thus, our results indicate that G4s might be drivers of local cSNV formation in cancer patients, while having a lesser effect on SNVs outside of cancer.

Recently, G4s have emerged as drivers of genomic plasticity, both through small- and large-scale genomic variations (4,77). Several different mechanisms have been described, which could explain how G4s cause elevated nucleotide substitution frequencies – rather than large-scale genomic instability – inside the PQS and the direct flanking base pairs. One possibility is that DNA polymerase errors during replication or repair processes at G4 loci contribute to elevated mutagenesis due to steric, G4 mediated inhibition (78). Error-prone polymerases might be engaged at slowed or stalled replication forks to ensure complete G4 replication, as specialized polymerases like eta, kappa, and Rev1 are essential for G4 stability – further increasing the density of localized genomic damages (26,78–80). Additionally, G-rich sequences, like G4 motifs, are prone to oxidative DNA damage, which can lead to mutations if not repaired properly (76). Lastly, more serious DNA damages, such as double-strand breaks associated with non-B DNA structures like G4s, and the subsequent DNA repair can be mutagenic (7,27). Notably, G4s and non-B DNA structures in general have been shown to trigger genomic instability through wrongfully stimulating DNA damage and repair responses which initially maintain genome integrity. Deviating DNA helix structures as a result of G4 formation can be recognized by structure-specific repair proteins and result in subsequent cleavage and repair, retaining the original sequence. This again allows for repeated G4 formation and cleavage, ultimately introducing a mutational pressure to prevent G4-formation (81,82).

While not exclusively resulting in point-mutations, one well-documented mechanism for G4-mediated genomic instability involves G4-enhanced formation of R-loops, that can spread into adjacent regions containing G4s (27). As G4s often occur clustered, this could lead to clusters of single-stranded R-loops in the flanking region of G4s that would be more susceptible to DNA damage. This observation could explain why cSNVs tend to accumulate in the immediate vicinity of G4s rather than within the G4 motif itself. It may also provide a possible explanation for the finding that windows containing clusters of G4s display a higher ratio of cSNVs compared to those with fewer G4s: If other G4s are within sufficient distance, R-loops might extend and open the chromatin, allowing additional R-loop formation in the adjacent G4s and further extension of single-stranded DNA regions. This would not be possible for solitary G4s. Nevertheless, while these mechanisms could explain how G4s drive genome plasticity through nucleotide variation, they predominantly substantiate the presence of SNVs within PQS or their immediate adjacent regions. They do not directly account for our observed elevation in cSNV-frequencies in G4-rich multi-kilobase pair windows. Ultimately, understanding the interplay between these mechanisms is crucial for elucidating the role of G4s and their flanking regions in genomic instability and disease development.

It is important to note that our correlative study design has a significant limitation: We cannot make a definitive assumption about the directionality of the correlation, i. e. whether cSNVs correlate with G4s (with G4s as drivers of nucleotide variation) or G4s correlate with cSNVs (with cSNVs providing more permissive conditions for the formation of G4-sequences). However, G4s hold various regulatory functions in transcription and translation (20), deregulated or ligand-induced G4-formation is associated with the activation of DNA-damage responses, implying that aberrant G4-formation triggers similar pathways as DNA-damage (83). Furthermore, G4s are highly susceptible to oxidative damage, as G-rich sequences have a low redox-potential and thus can act as ROS-sinks (84). Taken together, these findings point to a scenario in which G4s pre-exist and potentially function as initiators for the occurrence of cSNVs.

One possible explanation for the observed difference between cSNVs and dbSNVs could be system-wide alterations in cancer cells, which are often characterized by increased replication stress, altered DNA repair mechanisms or changes in chromatin structure (85,86). Other possible causes include altered chromatin remodeling, altered transcription landscapes or cancer-related environmental stressors impacting G4-formation or gene expression (87,88). All these factors could influence G4-formation- and -stability, potentially enhancing G4-mediated genomic instability and thus drive the association between G4s and cSNVs in cancer patients. Furthermore, cancer cells are subjected to high selection pressure due to their highly proliferative nature, favoring the acquisition of mutations that provide further growth advantages (89). As G4s are predominantly located in functional regions and cancer-genes, cellular changes resulting in the genome-wide stabilization of G4s could result in genomic instability in genomic regions which most likely confer growth advantages. In contrast, the selection pressure for proliferation-enhancing mutations is much lower in non-cancer cells – thereby providing a possible explanation for the observed differences in the correlation of cSNVs or dbSNVs and G4s.

Considering the observed striking differences in the spatial correlation of cSNVs and G4s across different cancer types, our results underscore the significance of stratifying patients based on their specific cancer diagnoses. It might even be applicable to sub-stratify patients with similar pathogenetic backgrounds by the location and abundance of chromatin-mapped G4s, as a recent study revealed G4-associated intratumor heterogeneity in breast cancer patient-derived tumor xenografts beyond current classifications (90).

This might be of significant relevance in the context of recent clinical trials evaluating the efficacy of G4-stabilizing ligands in cancer treatment. Notably, a Phase 1 clinical trial of the G4-stabilizing ligand CX-5461 (91), administered to patients with advanced solid tumors, demonstrated a 14% partial response rate in patients deficient in homologous recombination (HR), a critical DNA repair pathway, suggesting that the therapeutic efficacy of CX-5461 may largely rely on the induction of genomic instability and a compromised DNA damage response (92). While our study focused on the detection of SNVs in relation to G4s, it is highly probable that the patients we investigated experienced other forms of G4-mediated DNA damage, including insertions, deletions and double-strand breaks - as G4-mediated genomic instability is not limited to a specific type of DNA damage but rather has been observed to contribute to various types of DNA damage. By identifying cancer types with cSNV-landscapes predominantly associated with G4s, we can better understand which patients might respond more effectively to G4-stabilizing ligand therapies, which mostly rely on an increased genomic instability in cancer cells to slow or stop cancer progression and cell growth (83). In contrast, for cancer types that exhibit only a weak correlation between cSNVs and G4s, alternative therapeutic approaches may be more appropriate. This personalized approach has the potential to improve the effectiveness of G4-targeting therapies and minimize the risk of adverse effects for patients, ultimately leading to better patient outcomes.

Lastly, our findings suggest that high G4-content can serve as a useful prognostic marker for predicting the local density of cSNVs in cancer patients. The strong link between G4s and cSNV enrichment, especially in cancer-related genes, emphasizes the potential of G4s as a tool for predicting genomic regions more likely to harbor cancer-related mutations. Combining G4-content analysis with other genomic features could enhance the detection of genomic instability hotspots, helping to develop more precise diagnostic and treatment approaches for cancer patients. Most importantly, we observed a connection between the patient-specific G4-average in regions with cSNVs and individual survival times. This observation has significant implications for personalized cancer prognosis and treatment, as it suggests that analyzing the G4-average in regions with cSNVs could provide valuable insights into a patient's survival outlook. However, the substantial differences in G4-averages across cancer types necessitate further optimization before the G4-average can be used as a reliable prognostic marker. The development of cancer-specific G4-based models, particularly for subtypes with stronger G4-survival associations, is crucial for leveraging this genomic feature's prognostic potential. The model's relatively low concordance likely originates from the heterogeneous training data, which could be improved by focusing on patients with similar diagnoses. While G4-average analysis holds promise for personalized prognosis, rigorous validation accounting for inter-cancer variability is essential prior to clinical implementation. Our analysis thus serves as a foundation for future research to refine and improve the prognostic utility of G4-content, emphasizing the importance of careful data mining and cancer-specific tailoring in translating these observations into clinical practice.

In conclusion, our analysis provides a solid basis for further research into the role of G4s in cancer and the mechanisms by which they contribute to genomic instability. The observed correlation between cSNVs and G4s in cancer patients highlights the importance of G4s in cancer development and emphasizes the need for a deeper understanding of the molecular mechanisms involved. As personalized medicine advances, integrating G4-content analysis with other genomic features could lead to more precise identification of genomic instability hotspots and improved patient outcomes. Continued investigation of the causal relationship between G4s and cSNVs, as well as their influence on patient survival, will be crucial for harnessing the full potential of G4s as a therapeutic target in cancer therapy.

## Data Availability

The computational scripts, tools and methods employed in this study are fully documented and available: https://doi.org/10.5281/zenodo.8398567.
